# Off-label psychopharmacologic prescribing for children: History supports close clinical monitoring

**DOI:** 10.1186/1753-2000-2-24

**Published:** 2008-09-15

**Authors:** Julie M Zito, Albert T Derivan, Christopher J Kratochvil, Daniel J Safer, Joerg M Fegert, Laurence L Greenhill

**Affiliations:** 1 Pharmaceutical Health Services Research, School of Pharmacy, University of Maryland, Baltimore, Maryland, USA; 2 Psychiatry and Human Behavior, Thomas Jefferson University School of Medicine, Philadelphia, Pennsylvania, USA; 3 Psychopharmacology Research Center, University of Nebraska Medical Center, Omaha, Nebraska, USA; 4 Department of Psychiatry, Johns Hopkins Medical Institutions, Baltimore, Maryland, USA; 5 Department of Child and Adolescent Psychiatry/Psychotherapy, University Hospital Ulm, Germany; 6 Department of Psychiatry, Columbia University Medical Center, New York, New York, USA

## Abstract

The review presents pediatric adverse drug events from a historical perspective and focuses on selected safety issues associated with off-label use of medications for the psychiatric treatment of youth. Clinical monitoring procedures for major psychotropic drug classes are reviewed. Prior studies suggest that systematic treatment monitoring is warranted so as to both minimize risk of unexpected adverse events and exposures to ineffective treatments. Clinical trials to establish the efficacy and safety of drugs currently being used off-label in the pediatric population are needed. In the meantime, clinicians should consider the existing evidence-base for these drugs and institute close clinical monitoring.

## Background

Most medications are approved for marketing based on favorable benefit to risk assessments from clinical trial data in adults. Pediatric medical practice has been primarily off-label, i.e., permissible even though the drug was not specified for this age group, or indication in the product label approved by the Food and Drug Administration (FDA) [1]. Off-label use of a drug is a common practice representing approximately 50–75% of pediatric medication use [1]. In Europe, medication use may be characterized as either unlicensed, i.e. not approved for use in a particular age group, or off-label, i.e. outside the terms of their product license or marketing authorizations [2]. Occasionally, products not approved for use in children have statements declaring inadequate data or have warnings in their product label of potential dangers associated with pediatric use. Being off-label does not constitute a contraindication to the use of the product in children, so practitioners are free to prescribe the drug. Fost, a pediatric ethics expert, reminds clinicians that despite their frequent use, such off-label treatments may be perceived as "standard treatments" and lead individuals fearful of experimental treatments in clinical trials to prefer these inadequately evaluated but commonly used treatments [3].

A Medline search since 1990 and a review of clinical textbooks in Pediatrics [4], Pharmacology [5], and Child Psychopharmacology [6] were conducted to identify selected safety issues representing important concerns in medical treatment. This paper reviews the safety of off-label pediatric medications from two perspectives: an historical perspective that describes pediatric medication with established risks which were identified after many years, and a focused perspective on current psychopharmacologic treatment, assessing the need and expectations for adequate clinical monitoring. European experience with clinical monitoring is described briefly as a comparison in health systems predicated on a similar theoretical model of psychiatry.

### Historical examples from pediatric medicine

Off-label pediatric drug use has been based primarily on extrapolation of efficacy, dosing, administration and side effect profiles from adult studies. For treatments specific to youth, particularly to the neonate, the evidence is most often based on anecdote, case reports or open studies of clinical experience. Yet, the history of pediatric pharmacology is rich with examples illustrating that newly marketed drugs off-label for youth may have incomplete adverse event profiles that require widespread community utilization in such populations before uncommon or rare serious adverse events are known [7]. This drug information system does not well serve special populations, such as children. Several cases illustrate the risks even for commonly used and accepted treatments.

The use of oxygen therapy to improve breathing for babies in incubators was a widely accepted treatment as far back as the 1930s. In the 1940s, increases in dosage and length of exposure of oxygen were gradually accepted without safety research. The incidence of retrolental fibroplasia suddenly increased, followed by considerable debate in the literature over the suggestion that prematurity itself was responsible for the condition. Epidemiological data suggested that the increase in retrolental fibroplasia's adverse event rates following oxygen use varied by locale and practice [8]. Yet it was not until 1952, more than a decade later, that a definitive study linked increased oxygen use with the development of retrolental fibroplasia and blindness in premature babies [9].

The late '40s saw the introduction of chloramphenicol, an important new antibiotic with the promise of effectiveness in serious infections not controlled by available drugs. Within a decade, however, increased use resulted in the development of 'grey baby' syndrome in many of the infants so treated. This devastating and lethal illness of neonates, occurred due to inadequate enzymes to metabolize the drug to the glucuronide salt and then on insufficient renal filtration rate for excretion [10].

Recently marketed products also pose safety concerns for children. For example, propofol, a sedative-hypnotic, was marketed in 1989 in the U.S. and used for pre-anesthesia induction. Trial data in children from 1988 showed it had a 9% mortality rate in critically ill patients with upper respiratory tract infections compared with 4% for standard sedatives, but causality was not established [11]. Since then, propofol's use in pediatric intensive care units has been linked with 'propofol infusion syndrome'. This syndrome induces hypotension and metabolic acidosis, and produces a propofol metabolite that may induce toxicity [12] or predispose patients to sepsis [13]. In the summer of 2003, the FDA recommended a warning letter be sent to doctors based on adverse event reports from MedWatch (the FDA voluntary post-marketing surveillance reporting system). This experience illustrates that the original recommendations for dosage and rate of administration were not appropriate for all neonates and that the drug's usage in clinical trials could not be generalized to longer exposures or more rapid rates of titration in neonates treated in the community [14].

Pediatric drug safety issues might be viewed narrowly as simply the consequence of immature enzyme systems in the neonate. But the history of pharmacology proves this assumption wrong – elementary school age children can also be at increased risk of adverse events [15] and can experience problems distinctly different from adults treated with the same drug. A good illustration is tetracycline, a broad-spectrum antibiotic widely acclaimed and enthusiastically prescribed when it was introduced in 1955. However, it would take 8 years for a definitive paper to demonstrate that this antibiotic was responsible for hypoplasia and staining of the enamel of primary and secondary teeth [16]. Children are at risk starting from uterine exposure in the last trimester of pregnancy up to 8 years of age – the years of odontogenesis. In hindsight, the structure of a chemically altered microbial metabolite explains the loss of enamel through chelation of calcium ions. Before this safety issue was recognized, several million children were exposed to tetracycline with probably few of the cases justifying the selection and use of this drug.

Phenobarbital was introduced as an antiepileptic more than 90 years ago. Currently, its long-term use in children and adolescents is rarely justified because it is now known to increase the risk of adverse cognitive and behavioral events [17]. These effects include diminished intelligence [18] and behavioral problems e.g., misconduct and 'hyperactivity' [19]. Phenobarbital continues to be used for the control of simple febrile seizures and other seizures of obscure etiology [20] in children despite the fact that pronounced behavioral toxicity has been known for more than 25 years [21]. Pharmacoepidemiologic data on 4.3 million youth (0–17 years) from across the U.S. and with commercial health insurance illustrate this fact. In 2005, oral phenobarbital was dispensed to 0.025% of youth (2,649), which was 7.4 times more likely in children less than 2 years of age than their older counterparts [22].

Promethazine is a phenothiazine type antihistamine used in over-the-counter cough and cold products for the treatment of allergic symptoms. FDA's recent Public Health Advisory recommends avoiding use in children less than 2 years of age because of reports of serious and potentially life-threatening respiratory depression [23]. This report illustrates the gradual accrual of information for a cough and cold medication marketed since the late 1970's to update its safety profile [24].

This brief historical review of serious pitfalls in pediatric drug safety suggests the need for reassessing and updating the level of confidence required for prescribed drug use in children. This is true for general medical conditions, but is particularly true for the treatment of emotional and behavioral disorders. The reasons behind this specific emphasis include: 1) the rapid, expanded use of many drugs for psychotherapeutic purposes, both singly and in combination [25,26]; 2) the absence of current guidelines for prescribing off-label psychotropic drugs and the need to extend guidelines across physician specialties so that *both *pediatricians and child psychiatrists (and other clinical prescribers) follow appropriate standards of practice; 3) the absence of objective markers of emotional and behavioral conditions which can limit solid decision-making on the use of psychotropic medications; and 4) the need for close clinical monitoring and the engagement of parents and caregivers in such activities. The recent actions of the FDA and other regulatory bodies regarding antidepressant medication use in children make this need all the more salient.

### Historical examples from child mental health

The use of pemoline illustrates the challenges of drug safety for youth. While early evidence of hepatotoxicity in adults was recognized, the relatively low use in children meant that a long (unexamined) safety experience would accrue before a more definitive risk was recognized. After 21 years of modest usage in the treatment of attention-deficit/hyperactivity disorder (ADHD), liver toxicity including fatalities in youth were significantly associated [27]. Warnings were added in 1996 and a black box warning was added in 1999 as well as new requirements for written consent and biweekly liver enzyme monitoring. Unfortunately, little empirical evidence could be found that prescribers of pemoline were following this directive [28]. In May 2005, Abbott Laboratories announced their voluntary withdrawal of this drug from the U.S. market, and the FDA finally withdrew approval of generic pemoline in November 2005, a full 6 years after the drug was withdrawn in Canada [29].

### Current pediatric psychopharmacologic safety concerns

The psychotropic treatment of youth has expanded substantially since 1990, a relatively short time period from a population-based safety perspective [25]. In addition, several major drug classes [e.g., selective serotonin reuptake inhibitors (SSRIs) and atypical antipsychotics] represent novel compounds (new molecular entities) introduced, implying that much less information is known about their safety profile at the time of marketing [30]. Data on atypical antipsychotic adverse effects in large community-treated populations of adults are just beginning to emerge [31] and data on youth are even rarer [32]. Because off-label conditions increase the level of uncertainty regarding a drug's safety, Table 1 and Table 2 differentiate psychotropic drug use by their labeling status.

**Table 1:** Psychotropic drugs and FDA labeled psychiatric uses in youth.*

**Class, Subclass**	**Drug**	**Age Limits, yr.**	**Indication**
Stimulants			
	Amphetamines	3+	ADHD; Narcolepsy
	Methylphenidate	6+	ADHD; Narcolepsy
	Modafinil	16+	Narcolepsy
Antidepressants			
SSRI			
	Fluoxetine	8+	Depression
		7+	OCD
	Fluvoxamine	8+	OCD
	Sertraline	6+	OCD
TCA			
	Clomipramine	10+	OCD
	Doxepin	12+	Depression
	Impiramine	6+	Enuresis
		12+	Depression
Antipsychotics			
Conventional			
	Chlorpromazine	6 (mo)-12	Severe Behavior Problems; Psychosis
	Haloperidol	3+	Tourette's Disorder; Psychosis; Severe Behavioral Disorders
	Thiothixene	12+	Psychosis
	Loxapine	16+	Psychosis
	Molindone	12+	Psychosis
	Fluphenazine	12+	Psychosis
	Trifluperazine	12+	Psychosis
	Perphenazine	12+	Schizophrenia
	Pimozide	12+	Tourette's Disorder
	Prochlorperazine	2–12	Psychosis
	Thioridazine	2+	Schizophrenia
Atypical			
	Aripiprazole	13+	Schizophrenia
		10+	Acute and Mixed Mania
	Risperidone	10+	Acute and Mixed Mania;
		5–16	Irritability in Autism
		13+	Schizophrenia
Miscellaneous			
	Atomoxetine	6+	ADHD
	Chlordiazepoxide	6+	Anxiety
	Clorazepate	10+	Anxiety
	Desmopressin Oral	6+	Enuresis
	Diazepam	6 months +	Anxiety
	Flurazepam	15+	Insomnia
	Hydroxyzine	< 6	Anxiety
		> 6	Sedation
	Lithium Carbonate	12+	Manic Episodes
	Promethazine	2+	Sedation

*This information was current on March 12, 2008 based on the Physicians Desk Reference 2007 or FDA announcements. Readers should consult FDA guidelines for most current drug labeling.

**Table 2:** Common off-label uses of psychiatric drugs in U.S. youth.*

**Class and subclass**	**Drug**	**Off-label use**
Stimulants		
	Modafinil	ADHD
Antidepressants		
SSRI		
	Citalopram	Depression; Anxiety
	Duloxetine	Depression; Anxiety
	Escitalopram	Depression; Anxiety
	Paroxetine	Depression; Dysthymia; Anxiety; OCD
	Sertraline	Depression
Other		
	Bupropion	Depression; Anxiety ADHD
	Mirtazapine	Depression; Sleep
	Venlafaxine	Depression; Anxiety; ADHD
Antipsychotics		
Atypical		
	Clozapine	Psychosis; Bipolar, Behavioral and Tic Disorders; Schizophrenia < 16
	Olanzapine	Psychosis; Bipolar, Behavioral and Tic Disorders
	Quetiapine	Psychosis; Bipolar, Behavioral and Tic Disorders; Autism
	Ziprasidone	Psychosis; Bipolar, Behavioral and Tic Disorders; Autism
Anticonvulsant-Mood Stabilizers		
	Divalproex	Bipolar Disorder; Aggression
	Gabapentin	Bipolar Disorder
	Lamotrigine	Bipolar Disorder; Depression
	Oxcarbazepine	Bipolar Disorder; Aggression
Alpha-Agonists		
	Clonidine	Sleep; ADHD; Aggression; Autism; Tourette's
	Guanfacine	Sleep; ADHD

*This information was derived from WH Green [6]

Changes in anticonvulsant pediatric usage in the past decade are largely attributed to their increased use for psychotherapeutic purposes, specifically as mood stabilizers [33]. Fortunately, adverse events associated with anticonvulsant use in children have been widely studied, largely as a result of the need for better treatments for seizure disorders. Valproic acid was a welcome addition to the anticonvulsant market in 1978. But soon after marketing, case reports of serious events in children began to emerge. Dreifuss and colleagues reviewed all U.S. reports of fatal hepatic dysfunction received by the manufacturer in the first six years of marketing. The large majority of these reports (86.5%) involved use of another anticonvulsant in addition to valproate. Age and combination use were found to be the greatest risk factors for fatal hepatotoxicity: children less than 2 receiving valproate as polytherapy had a 20-fold greater risk compared to older ages [34]. While early data were narrowly interpreted as a risk associated with immature liver enzyme metabolism of very young children, subsequent reports revealed an elevated risk among older children as well (3–10 years olds, especially those on polytherapy) [35]. A review of the world literature revealed that more than 90% of the approximately 100 fatalities occurred in patients less than 20 years of age [35]. The risk of hepatotoxicity in youth treated with multiple anticonvulsants which rely on liver enzyme systems for their metabolism suggests that neuropsychiatric concomitant drug regimens which also rely on liver metabolism should be treated with special caution.

### Psychotropic adverse events in youth

Table 3 shows selected major adverse events for both recently approved and off-label psychiatric drug usage with suggested safety surveillance monitoring in children and adolescents. Whether the selected medications are newly marketed or off-label, surveillance is appropriate because exposed youth populations have been limited relative to adult populations [25]. Klein has advocated for more rigorous post-marketing surveillance of adverse events in psychiatry by using large commercial datasets that would permit analysis of adverse event incidence rates [36], an advantage over the existing FDA MedWatch system. In the addition to a revised safety infrastructure, it is critical that prescribing physicians perform careful, systematic clinical monitoring to avoid unnecessary risk [6].

**Table 3:** Suggested adverse event monitoring for selected medications used to treat labeled and unlabeled psychiatric indications in children and adolescents

**Drug Class**	**Drug**	**Adverse Events**	**Comment; Monitoring tool**
Alpha-Agonists	Clonidine Guanfacine	Bradycardia; Hypotention; Heart block	Rule out congenital heart disease; Blood pressure and heart rate
Stimulants	Amphetamines	Serious cardiovascular risk [59]	Blood pressure and heart rate; ECG where there is a question of congenital heart disease
Anticonvulsant-Mood Stabilizer	Divalproex; Valproic acid	Polycystic ovaries in girls; malformation rate of 11.1% compared with 3.1% in non-drug exposed fetuses [62]; hepatotoxicity [63]; pancreatitis [64,65]	Discuss risks and provide written information before initiating therapy; girls of child-bearing age should be counseled regarding birth control. Close laboratory monitoring of liver enzymes & coagulation tests in the first 6 months; clinical monitoring for vomiting and apathy; blood levels
	Lamotrigine	Rash requiring hospitalization, possible Stevens Johnson Syndrome or hypersensitivity syndrome; serum concentrations doubled when divalproex was added in adjunctive treatment of epilepsy.	Indication in those younger than 16 is restricted to Lennox Gastaut Syndrome. Black box warning for potentially life threatening rashes
Antidepressants	SSRIs	Activation syndrome, suicidality	A written diary by the parent of target symptoms and selected adverse events is useful. Regular contact to review information when drug or dose is initiated or changed. Monitor side effects and response regularly
	TCAs	Dose-dependent cardiac conduction delays; asystole	Baseline and follow-up ECG at therapeutic dose, blood levels.
	Bupropion	Dose-dependent risk of seizure	Consider alternatives in youth with a history of seizure disorders or bulimia
Atypical Antipsychotics	Olanzapine Risperidone Quetiapine Clozapine Ziprasidone	Relatively greater weight gain in youths than in adults Extrapyramidal Side Effects (EPS) Hyperprolactinaemia Possible Hyperthyroidism (Quetiapine)	Baseline and repeat weight, height and waist circumference, serum fasting lipid and hepatic enzyme levels, thyroid panel (for quetiapine). Fasting glucose level monitoring for the risk of diabetes; diet and exercise management. Monitor quarterly or as indicated for movement disorders with the Abnormal Involuntary Movement Scale (AIMS). Prolactin blood level monitoring in the presence of abnormal sexual signs and symptoms.
Lithium	Lithium	Thyroid abnormalities; nephrotoxicity; renal concentration diminution; lithium toxicity	Lithium levels, baseline thyroid panel, serum creatinine and urinalysis. Repeat periodically, and when dose or regimen changes or symptoms suggest toxicity.

Of the SSRIs, only fluoxetine has been shown to be effective for the treatment of depression in children and adolescents [37,38]. However, the occurrence of suicidal events in community-treated adults and in clinical trials of adolescents which appeared shortly after the launch of this new class of antidepressants [39,40] raised concerns, but these were left unresolved by the FDA. Regulatory events related to the pediatric use of SSRIs and suicidal risks in youth were initiated by Medicines and Healthcare products Regulatory Agency (MHRA) in U.K. in 2003 and rapidly produced a similar scenario in the U.S. in 2004, culminating with a black box warning for *all *3 classes of antidepressants, namely SSRIs, tricyclic antidepressants (TCAs) and Other antidepressants [41].

Other adverse events are more common with antidepressants and may be useful in identifying who is at risk for suicidal thoughts or attempts. For example, activation was highlighted in the Hammad meta-analysis in association with suicidal ideation or behavior [42]. Unfortunately, the timing of this symptom was not available in relation to the adverse suicidal events and thus could not be identified as a risk factor. An added confusion is the absence of procedures for identifying adverse events in a consistent manner across companies (for trials) and across voluntary reports in the Medical Dictionary for Regulatory Activities (MedDRA), the data dictionary for MedWatch [43]. Thus, activation/agitation/hostility has multiple descriptors in clinical trials, including hyperkinesis. These events are more frequent in clinical trials with children than with adults. For example, in SSRI pediatric trial data, the average frequency of activation or agitation in children was 10–15% [44]. Recently, the FDA announced that meta-analysis of adverse psychiatric events in clinical trials of anticonvulsants for seizure, psychiatric disorders and other conditions were significantly greater for drug-treated vs. placebo-treated subjects (0.4% vs. 0.22%). Whether meta-analysis of data with incomplete historical data on risk factors such as psychiatric history is adequate to substantiate increased psychiatric symptoms in major anticonvulsants for seizure disorder deserves further assessment [45]. In summary, the lack of standardization of adverse event reporting in clinical trials [46] limits comparative safety assessments from trials and argues for improved adverse drug event monitoring in clinical trials and for more prospective studies of adverse events in the post-marketing surveillance phase of drug development and appraisal.

### Systematic clinical monitoring for psychotropic drug safety

The growing use of concomitant psychotropics in U.S. children [47,48] raises special concerns. Such use is generally off-label and often without systematic study to assure either efficacy or safety. To improve the confidence of prescribing physicians in the safety of monotherapy as well as combination pharmacotherapy, regular monitoring is recommended. Monitoring refers to collecting and organizing information systematically with respect to time and relevance to the issues of concern. Information should be relevant to potential adverse drug events, effectiveness and satisfaction so that systematic monitoring is targeted to serious adverse events which are drug-specific, practical and timely. For example, for atypical antipsychotics, before treatment is initiated baseline physical measures should include body weight [measured as body mass index (BMI)], liver function tests and lipid measures so that subsequent treatment-emergent events can be more accurately associated with drug exposure [49].

### European standards for psychotropic drug safety

Most European country health care systems are substantially different than that of the U.S. European health insurance and access to care is usually available for nearly everyone either in state run systems or by state regulated health insurance companies. Despite this high standard for provision of care, many aspects of drug treatment safety are still neglected. Collecting safety information from health insurance data and networks to monitor and report adverse events could be easily regulated and implemented on a national level but still these initiatives rely on the activities of different insurance companies as shown in a recent report of the Gmünder Ersatzkasse, a statutory insurance company in Germany [50].

At the European Union level, The European Agency for the Evaluation of Medicinal Products (EMEA) is responsible for the implementation of the European Risk Management Strategy (ERMS) [51]. This strategy focuses on harmonizing European community legislation with respect to drug safety and thereby strengthening the European Union Drug Regulatory Authorities (EUDRA) vigilance, the population-based EU safety database. There are plans to introduce a special Eudra Vigilance Datawarehouse and Analysis System to enhance safety surveillance. In addition to spontaneous reporting of adverse event systems, a network of centers for pharmacoepidemiology and pharmacovigliance is planned which should facilitate the conduct of multicenter studies or authorize safety topics which fall under the European Network of Centres for Pharmacoepidemiology and Pharmacovigilance (ENCePP) and is a major aim of the EMEA.

At the country level, in German adult psychiatry a therapeutic drug monitoring network (TDM) was established [52-54]. Supported by a research grant after preparatory work by the commission on developmental psychopharmacology from the German professional societies in child and adolescent psychiatry in 2008, a child psychiatric TDM network was founded [55]. This therapeutic drug monitoring network comprises the measurement of plasma or serum levels and the documentation of clinical effectiveness and unwanted side effects. Therapeutic drug monitoring thus is aiming at defining therapeutic ranges of plasma or serum levels in order to maximize clinical effects while minimizing the risk of side effects or toxicity, particularly in high risk populations e.g., the developing child. Pilot work showing the high variation of plasma levels of atypical neuroleptics in children has been published [56]. The general need for this network was described by Gerlach et al. in 2006 [57], and has been accepted by the boards of the three professional societies in Germany.

In cases of inpatient treatment with psychotropic drugs, the German insurance companies pay for therapeutic drug monitoring as a measure of quality assurance in the field of pediatric psychopharmacology. In the Future the TDM model might be extended to large U.S practices using simplified validated adverse drug event monitoring. For example, in The Child and Adolescent Psychiatry Trials Network (CAPTN), the practice-based research network in child psychiatry, the pilot study of Pediatric Adverse Events Rating Scale (PAERS) [58] could be further validated by applying European TDM established findings on plasma level-side effect related data to U.S. youth populations.

At the most global level, the World Health Organization (WHO) promotes an international drug monitoring program which started operating in 1968. Currently, 86 countries participate in that program. Reported cases are forwarded from national pharmacovigilance centers to the WHO collaborating center for international drug monitoring in Uppsala Sweden. The case reports are stored in the Adverse Drug Reaction (ADR) database of the WHO which is the most comprehensive source of international ADR information. This time-honored system notwithstanding, there are significant challenges to improve the probability of finding serious and rare events in youth and to rule out long-term adverse effects on development. The encouraging signs of renewed efforts in the European Union collaborations are further aided by the advent of powerful computing systems and suggest that psychopharmacologic drug safety in children is progressing.

### Recommended baseline and ongoing monitoring of children and adolescents

A comprehensive assessment of health status (rating of symptoms and impairment) should be conducted **before **introducing psychotropic medications, whether utilized for labeled or off-label uses [6]. A comprehensive assessment at baseline includes physical measures such as pulse, respiration rate and blood pressure. Regular assessment of growth over time using standardized growth charts is recommended, including measures of height, weight (calculation of BMI) and with medications where weight gain is of concern waist circumference. Depending on the pharmacotherapy, a laboratory panel including complete blood count, urinalysis, blood urea nitrogen (BUN) level, serum electrolytes and liver function tests may be indicated. Such data would lessen post hoc conjecture regarding underlying physical abnormalities and the attribution of emergent adverse drug events. More importantly, it could improve the close monitoring of preexisting abnormal lab values or of organ function and lead to earlier interventions to reduce risks associated with drug-related events or drug interactions. Which laboratory assessments are indicated depends upon presenting symptoms as well as the selection of medication to be initiated. In addition, an electrocardiogram (ECG) may be appropriate when there is concern about potential changes in cardiac conduction, such as when a TCA is initiated. By establishing a baseline battery of physical health status, subsequent changes can be accurately assessed in terms of treatment-emergent adverse drug events.

The rationale for drug class-specific monitoring includes the following:

• Amphetamines. The growth in use of amphetamines since the marketing of Adderall^® ^is substantial with as much as half of U.S. stimulant use in youth now representing exposure to amphetamine salts rather than to methylphenidate. Consequently, recent concerns about cardiac risks from FDA analysis of MedWatch data sparked controversy [59] and Canadian agency reports of cardiac deaths raised a similar concern [60]. Until the issue is laid to rest, the value of baseline cardiac assessment to rule out the likelihood of cardiac abnormalities may be prudent [6].

• Alpha-Agonists. Clonidine and guanfacine were approved for adult treatment of hypertension. Since 1987, these drugs have been used off-label in pediatrics for the treatment of ADHD, to reduce stimulant rebound and induce sleep. Baseline evidence of cardiovascular health status is useful to permit adverse symptoms following drug initiation to be linked to the medication [6] and to avoid use in those with congenital cardiac anomalies.

#### • Anticonvulsant-Mood Stabilizers

∘ Divalproex and Valproic Acid. Valproate treatment initiated in women before the age of 20 had significantly increased risk of polycystic ovaries [61]. In addition, the occurrence of an 11.1% malformation rate in drug-treated compared with 3.1% in non-drug exposed fetuses has been reported [62]. Ongoing reports of hepatotoxicity even in youth older than 2 years of age and particularly in those with concomitant drugs that are liver metabolized warrant attention [63] as well as pancreatitis among youth with chronic exposure [64,65].

∘ Lamotrigine has been reported to have a higher risk of rash in children than adults [66]. Trial data demonstrated Stevens Johnson Syndrome, a serious rash often requiring hospitalization, and hypersensitivity syndrome occurred in 1% of children and in 0.3% of adults. Serum concentrations doubled when divalproex was added in adjunctive treatment of epilepsy.

#### • Antidepressants

∘ SSRI suicidality (ideation, attempts) was noted in an average of 4% of of children and adolescents treated with antidepressants in clinical trials reviewed by regulatory agencies [67]; other psychiatric adverse effects e.g., behavioral disinhibition, emotionality, activation, irritability, agitation have been found in up to 25% of children treated with SSRIs [68]; psychiatric adverse effects are more common in depressed youth less than 12 years old than in adolescents [41].

∘ TCA Dose-dependent cardiac conduction delays; asystole in high dose [6].

∘ Bupropion. The risk of dose-dependent seizures may suggest use of an alternative antidepressant to treat youth with a history of seizure disorder or bulimia.

• Atypical Antipsychotics. Relatively greater weight gain develops in youth than in adults [69] so that baseline and repeat weight, height and waist circumference should be measured. Because of the risk of metabolic syndrome, fasting glucose level monitoring for the onset of diabetes is warranted [49] as well as liver function and lipid tests. Diet and exercise management are useful, in light of the increased risk of weight gain. To assess adverse effects in clinical practice settings, a revised computerized version of the NIMH-developed DOTES psychopharmacologic monitoring scale was used. Extrapyramidal side effects including rigidity, tremor, and dystonia were seen in 5% to 15% of youth treated with olanzapine as well as risperidone [70].

• Lithium has a narrow therapeutic range which emphasizes the importance of educating parents and youth as to the need for adequate hydration and risks of exposure to situations where excessive sweating may occur (i.e. participating in sports, spending time in the heat outdoors), or if the child experiences significant diarrhea, in order to avoid toxicity. Baseline and repeat assessment of thyroid function as well as kidney function during use is recommended [71]. Laboratory assessment of lithium levels is helpful to avoid toxicity.

#### • Miscellaneous

∘ Desmopressin. Recently, an FDA alert warned of severe hyponatremia and seizures in children treated with intranasal formulations of desmopressin (DDAVP) for primary nocturnal enuresis. The nasal product is no longer indicated for primary nocturnal enuresis and oral formulations should not be used during acute illnesses which may lead to fluid and/or electrolyte imbalance [72].

### Periodic and ongoing monitoring for safety and effectiveness

After initiating drug therapy, safety assessments at regular intervals are useful to observe the ongoing impact of medication use (Table 3). Column 4 lists specific monitoring suggestions. More detailed schedules for monitoring can be found in Correll and Carlson [49]. Dose adjustments and the addition or withdrawal of concomitant drug therapy can be occasions for biological status checks with laboratory assessments and vital signs, in addition to assessing drug-specific adverse events.

School performance and social development are measures of the effectiveness of treatment on overall functioning, and the patient's or parent's report of adherence is vital to this assessment and may in part reflect the level of satisfaction. When cognitive or emotional symptoms show a lack of improvement or deterioration, an assessment of the temporal pattern of drug usage, dosage change and potential drug-drug interactions can assist in establishing whether behavioral toxicity, i.e. iatrogenic psychiatric symptoms, is a likely explanation. This could lead to the need for discontinuation of the psychotropic medication responsible for behavioral adverse events.

## Discussion

This selective review of pediatric medical and psychiatric drug usage illustrates the role of clinical monitoring as a routine aspect of post-marketing surveillance. Distinctions between off-label and labeled indications indicate that the majority of pediatric psychotropic use is off-label and supports close monitoring to assure adequate safety. Until all drugs are properly studied in the populations for which they are being used, it will be necessary for practitioners to prescribe off-label drugs. Additionally, it is sometimes necessary to utilize products that have uncertain efficacy and unresolved safety questions, even when such issues raise serious ethical and clinical considerations. The particular medical circumstances will dictate what clinical criteria and monitoring are most appropriate. An adequate diagnostic assessment and the establishment of sound baseline data are always mandatory. In addition, particular attention must be paid to the ethical considerations generated by clinical decisions to use off-label treatments, particularly where the evidence of efficacy is weak or anecdotal, and safety signals are unresolved. We believe the 1979 Belmont Report on ethical guidelines for the protection of human subjects of biomedical and behavioral research with its associated principles could provide an ethical framework for off-label usage in children in clinical practice [73]. Examining the issue of off-label use from the perspective of child-patient, parent-caregiver and prescribing physician offers a sound approach to the application of these principles. Armed with a better sense of history and ethics, practitioners in pediatrics and child psychiatry can provide safer treatments as we build a stronger evidence base for their use.

## Conclusion

This historical review presented examples of serious pediatric drug safety problems in the post-marketing phase of utilization and identified off-label psychiatric use. As a broad survey of pediatric psychiatric pharmacotherapy, it provides evidence to support changes in the way we monitor newly approved or off-label drugs for emotional and behavioral conditions in youth. Since there is less certainty about the outcome of these medications in children and adolescents (particularly in concomitant drug regimens), close clinical monitoring is critical to detect adverse physical and mental changes as well as to ascertain if there is ongoing reduction of symptoms and improved functioning. This approach would minimize ineffective treatments that expose youth to drugs of uncertain or unknown risks.

## Authors' contributions

JMZ, ATD, CJK, and LLG contributed to the conception and design; JMZ, ATD, DJS, CJK, JMF were involved in drafting, revising and interpreting the review. All authors read and approved the version to be published.
